# The tyrosine-kinase inhibitor sunitinib targets vascular endothelial (VE)-cadherin: a marker of response to antitumoural treatment in metastatic renal cell carcinoma

**DOI:** 10.1038/s41416-018-0054-5

**Published:** 2018-03-22

**Authors:** Helena Polena, Julie Creuzet, Maeva Dufies, Adama Sidibé, Abir Khalil-Mgharbel, Aude Salomon, Alban Deroux, Jean-Louis Quesada, Caroline Roelants, Odile Filhol, Claude Cochet, Ellen Blanc, Céline Ferlay-Segura, Delphine Borchiellini, Jean-Marc Ferrero, Bernard Escudier, Sylvie Négrier, Gilles Pages, Isabelle Vilgrain

**Affiliations:** 1 Univ-Grenoble Alpes, INSERM, CNRS, BIG-BCI Biology of Cancer and Infection, Grenoble, F- 38054 France; 2 Biomedical Department, Centre Scientifique de Monaco, Monaco, Monaco; 3 Grenoble University Hospital, Division of Internal Medicine, Grenoble, F-38043 France; 4 INSERM, Unité 003, Clinical Investigation Center, Grenoble University Hospital, Grenoble, F-38043 France; 5 Unicancer, Centre de Lutte contre Le Cancer Léon Bérard, Lyon, F-69008 France; 6 Department of Clinical Research, Innovation and Statistics, Centre Antoine Lacassagne, Nice, F-06000 France; 7 Gustave Roussy Cancer Campus, Grand Paris, Villejuif, F-94800 France; 8 University of Nice Sophia Antipolis, Institute for Research on Cancer and Aging of Nice, CNRS UMR 7284, INSERM U1081, Centre Antoine Lacassagne, Nice, F-06107 France

**Keywords:** Tumour biomarkers, Predictive markers

## Abstract

**Background:**

Vascular endothelial (VE)-cadherin is an endothelial cell-specific protein responsible for endothelium integrity. Its adhesive properties are regulated by post-translational processing, such as tyrosine phosphorylation at site Y^685^ in its cytoplasmic domain, and cleavage of its extracellular domain (sVE). In hormone-refractory metastatic breast cancer, we recently demonstrated that sVE levels correlate to poor survival. In the present study, we determine whether kidney cancer therapies had an effect on VE-cadherin structural modifications and their clinical interest to monitor patient outcome.

**Methods:**

The effects of kidney cancer biotherapies were tested on an endothelial monolayer model mimicking the endothelium lining blood vessels and on a homotypic and heterotypic 3D cell model mimicking tumour growth. sVE was quantified by ELISA in renal cell carcinoma patients initiating sunitinib (48 patients) or bevacizumab (83 patients) in the first-line metastatic setting (SUVEGIL and TORAVA trials).

**Results:**

Human VE-cadherin is a direct target for sunitinib which inhibits its VEGF-induced phosphorylation and cleavage on endothelial monolayer and endothelial cell migration in the 3D model. The tumour cell environment modulates VE-cadherin functions through MMPs and VEGF. We demonstrate the presence of soluble VE-cadherin in the sera of mRCC patients (*n* = 131) which level at baseline, is higher than in a healthy donor group (*n* = 96). Analysis of sVE level after 4 weeks of treatment showed that a decrease in sVE level discriminates the responders vs. non-responders to sunitinib, but not bevacizumab.

**Conclusions:**

These data highlight the interest for the sVE bioassay in future follow-up of cancer patients treated with targeted therapies such as tyrosine-kinase inhibitors.

## Introduction

Renal cell carcinoma (RCC) represents the 12th most frequent type of cancer and the 2% of all adult malignancies.^1^ Between 25 and 30% of the patients will develop metastatic renal cell carcinoma (mRCC) by the time they are diagnosed. Over the last 10 years three classes of targeted therapies have been developed, to treat mRCC. The first is multi-targeted tyrosine-kinase inhibitors (TKI) that include sorafenib, axitinib, pazopanib, and sunitinib. The second class is the mammalian target of rapamycin (mTOR) complex-1 kinase inhibitors represented by temsirolimus (TEM), everolimus. The third class is the humanised anti-vascular endothelial growth factor (VEGF) monoclonal antibody, bevacizumab (BEV), and interferon 2α (IFN).^2,3^

Since its approval in 2006 sunitinib (SUT) is a standard of care in the first line treatment of mRCC that has allowed an improved outcome.^4–7^ SUT is a multi-target TKI which belongs to the class of anti-angiogenic therapies that target the VEGF-signalling pathway.^8^ Despite significant improvements in survival, several reports have described acquired resistance to anti-angiogenic TKI after 6 months to 3 years of disease control.^9–12^ Thus, there is an urgent need for potential predictive biomarkers that could stratify patients who would benefit from treatment with SUT.

RCC is a highly angiogenic cancer. Angiogenesis involves the remodeling of endothelial cells (ECs), which acquire cell–cell contacts by homophilic interaction of vascular endothelial (VE)-cadherin molecules expressed by neighboring cells.^13^ In normal healthy vessels ECs are tightly bound to each other, while in angiogenic vessels in tumours, ECs are stimulated by cytokines and growth factors in a process that leads to cell–cell destabilisation.^14^ Several cellular pathways are involved in these processes through VE-cadherin targeting. Indeed, we have previously shown that the VEGF-induced-VE-cadherin phosphorylation contributes to the inhibition of the adhesive properties of ECs.^15^ This event occurs in vivo in mice in highly vascularised organs such as ovaries and uterus, during the angiogenic switch induced by hormones.^16–18^ Another mechanism involved in ECs destabilisation is the cleavage of the extracellular domain of VE-cadherin upon cytokines challenge such as TNFα^19^ and VEGF.^20^ Of major importance, a soluble form of VE-cadherin (sVE) has been detected in the bloodstream of patients with several diseases^17,18^ including breast cancer. Indeed, in metastatic breast cancer, we recently demonstrated that sVE was an independent prognostic factor for both progression-free survival and overall survival.^21^

The aim of the present study was to determine whether anti-angiogenic treatment commonly used for the clinical care of mRCC had an effect on VE-cadherin post-translational modifications (i.e., phosphorylation and cleavage) and if so, whether such modifications were helpful to monitor patient outcome.

## Materials and methods

### Reagents

Recombinant human VEGF 165 was obtained from PromCell (Rocky Hill, NJ, USA). Sunitinib, temsirolimus, interferon 2αΑ, irinotecan, SN38 (the active metabolite of irinotecan), poly-2-hydroxyethyl methacrylate (polyHEMA), sodium orthovanadate, and gelatin were purchased from Sigma-Aldrich (St Quentin Fallavier, France). CellTracker Green, collagen, Enhanced chemiluminescence detection reagents, the micro–bicinchoninic acid (micro-BCA) protein assay reagent kit were purchased from ThermoFisher Scientific (Courtaboeuf, France). Nitrocellulose was obtained from Schleicher & Schuell BioScience (Perkin-Elmer Life Science, FRANCE).

### Antibodies

We used the following commercially available antibodies including the mouse monoclonal antibody to human VE-cadherin (clone BV9) (Santa Cruz Biotechnology, SantaCruz, CA, USA), the monoclonal anti-phosphotyrosine antibody (clone 4G10) (Millipore Paris, France), the rabbit anti phosphoY^685^-VE-cadherin antibody (produced in the laboratory) and characterised in refs.^17,18^ and the mouse polyclonal anti–β actin (clone AC-15) (Sigma-Aldrich, St Quentin-Fallavier, France). As secondary antibodies, we used the horseradish peroxidase-conjugated goat anti-mouse and anti-goat immonoglobulin G antibodies (Bio-Rad Laboratories, Marnes la Coquette, France).

### Cell culture

#### Endothelial cells

Human Umbilical Vein Endothelial Cells (HUVECs) were grown and analysed as described by Vilgrain et al.^20^

#### Renal cells

The renal carcinoma-derived cell line 786-O from American Type Culture Collection (ATCC, Manassas, USA) was grown in RPMI medium supplemented with 10% FBS, 100 U/mL penicillin, 100 µg/mL streptomycin and 0.25 µg/mL amphotericin. Conditioned media from 10 cm dishes (8 × 10^6^ cells) were collected after serum starvation, centrifuged at 14,000 r.p.m. to discard flotting cells, and concentrated on Centriprep Centrifugal Filter Units with an Ultracel YM-30 membrane (SODIPRO, Echirolles, France) and kept at −80 °C before use.

#### Zymography

Zymograms were performed as described in Lê et al.^22^ using bovine gelatin (final concentration 1 mg/mL). Conditioned media from 786-O RCC cells were loaded onto a 12% acrylamide SDS-PAGE. After electrophoresis at 0.02 A for 2 h 30 min in Tris/HCl 25 mM glycine 192 mM buffer, the gelatin gels were then successively washed at room temperature (RT) under agitation twice in 100 mL 2.5% Triton X-100 to remove SDS for 30 min, then in 50 mM Tris/HCL, pH 8.5 containing 200 mM NaCl, 10 mM CaCl_2_ for 15 min and overnight (ON) in the same buffer at 37 °C. After three washing steps of 10 min in water, gels were successively stained with Coomassie blue for 1 h at RT before being destained in 100 mL 20% methanol 10% acetic acid for 30 min and then in water.

#### **Preparation of cell extracts, electrophoresis, and immunoblotting**

Cell lysates, and immunoblots were prepared and analysed as described previously by Vilgrain et al.^20^

#### Generation of spheroids

Confluent monolayers ECs were incubated for 30 min with 10 μM of the CellTracker Reagent. After 4 h of incubation, ECs were trypsinised and suspended either alone or with 786-O RCC cells (1:1) onto poly-HEMA-coated plates (20 mg/mL). Under these conditions all suspended cells contributed to the formation of a single spheroid. After 72 h of culture, the spheroids were collected, transferred onto a collagen gel (4 mg/mL), and stimulated with either VEGF alone (50 ng/mL), or in combination with mRCC therapies. RCC and ECs migration from the spheroid was imaged with a Zeiss AxioObserver Z1. After 24 h of culture, the position of each EC (*n* = 500) was manually recorded using the ImageJ Cell^_^Counter plugin (http://rsbweb.nih.gov/ij/plugins/cell-counter.html) (http://imagej.nih.gov/ij/). *X* and *Y*-values were then imported in a customised Python (https://www.python.org) script, which we created to calculate the Euclidean distance of each cell to the centre of the spheroid and the mean distance of the cells to the centre (Supplementary Figure S1).

#### Enzyme linked immunosorbent assay (ELISA)

ELISA for soluble VE-cadherin was performed as described in Vilgrain et al.^20^

### Patients’ blood samples

#### Healthy donors (HD)

Sera from healthy donors eligible to give their blood were collected at “Etablissement Français du Sang” (EFS, Grenoble, France) and kept frozen at −80 °C until use. The study protocol was approved by the EFS and written informed consent was obtained from each participant.

#### Patients with metastatic renal cell cancer

Sera from patients with metastatic renal cell cancer receiving SUT or TEM + BEV or BEV + IFN were obtained from 2 clinical trials (TORAVA and SUVEGIL) approved by local ethics committee. Written informed consent was obtained from each patient before enrollment. Patients’ characteristics are described in Supplementary Table 1.

#### TORAVA. (clinicaltrials.gov NCT00619268—sponsor: Centre Léon Bérard, Lyon, France)

Patients’recruitment and data-collection methods of the TORAVA trial were described in (Negrier et al.^44^). Blood samples were collected at three different time points: at baseline prior to treatment (Day 0), 2 weeks (Day 15), and 5–6 weeks (Day 40) after the beginning of the treatment. Samples were stored at −196 °C in liquid nitrogen after collection in the Biological resource centre BB-0033-00050 of the Centre Léon Bérard, Lyon, France.

#### SUVEGIL. (clinicaltrials.gov NCT00943839– sponsor: Centre Antoine Lacassagne, Nice, France)

Each cycle lasted 6 weeks in the absence of disease progression or unacceptable toxicity and was organised in the protocol of four consecutive weeks of oral administration of sunitinib malate (50 mg/day) followed by 2 weeks of rest. Blood samples were collected before the treatment and at the end of the fourth week of SUT treatment.^23^

### Statistical analysis

#### For cell analysis

All of the experiments were repeated at least three times. Western blot bands have been analysed to densitometry using Image J software and data are expressed in arbitrary units as the mean ± SD of at least three identical experiments (same number of cells in each dishes). Student’s *t*-test was used to compare the means of data from two experimental groups. One-way ANOVA was used when three or more experimental groups were compared, and analysis of significance was performed using Tukey’s range test (*P < 0.05; **P < 0.01; ***P < 0.001). For all tests, *P*-values ≤0.05 were considered significant.

#### For sVE analysis in blood sample

The non-parametric Mann–Whitney *U*-test was used to compare sVE levels between mRCC patients and the HD group. Wilcoxon and Fisher Exact tests were used to evaluate the relationship between sVE levels and clinical response to treatment. Progression-free survival was defined as the time from inclusion to the date of first documentation of progression or the date of death or date of last follow-up. Survival data were assessed by the Kaplan–Meier method. Statistical analysis was performed using Stata release 11.0 (StataCorp, College Station, TX)—PC software. For all tests, *P-*values <0.05 were considered statistically significant.

## Results

### VE-cadherin is a target for Sunitinib

**Fig. 1:**
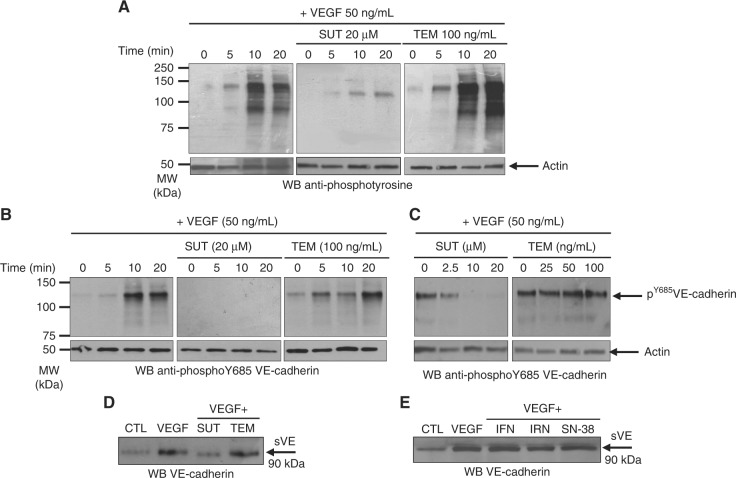
Sunitinib impairs VEGF-induced VE-cadherin tyrosine phosphorylation and cleavage: primary ECs were grown 3–4 days to reach confluency (3 × 10^6^ cells). After 3 h of serum starvation, ECs were stimulated by VEGF alone (50 ng/mL) or in the presence of SUT (20 μM) or TEM (50 ng/mL) during indicated times. Lysates were analysed by SDS-PAGE on 10% polyacrylamide gels and western blotting (5 μg of total protein lysate/lane). **a** Analysis of phosphotyrosine-containing protein pattern using the anti-phosphotyrosine antibody 4G10. **b**, **c** Analysis of phospho-Y^685^VE-cadherin **d**. Conditioned media from untreated ECs (CTL), or VEGF-stimulated-ECs or VEGF-stimulated-ECs treated with SUT or TEM for 20 min (same amount of cells in each condition) were collected, centrifuged to discard floating cells, and concentrated on Centriprep Centrifugal Filter Units with an Ultracel YM-30 membrane. Volume of 5 μL of concentrated medium of each condition was analysed by SDS-PAGE and western blotting using the monoclonal anti-VE-cadherin antibody (BV9 clone). **e** Same experiment as in **d** with VEGF-treated ECs in the presence of either INFα2 A (7.5 ng/mL), Irinotecan (IRN, 10 μM) and SN-38 (10 μM). Results are representative of three independent experiments

**Fig. 2:**
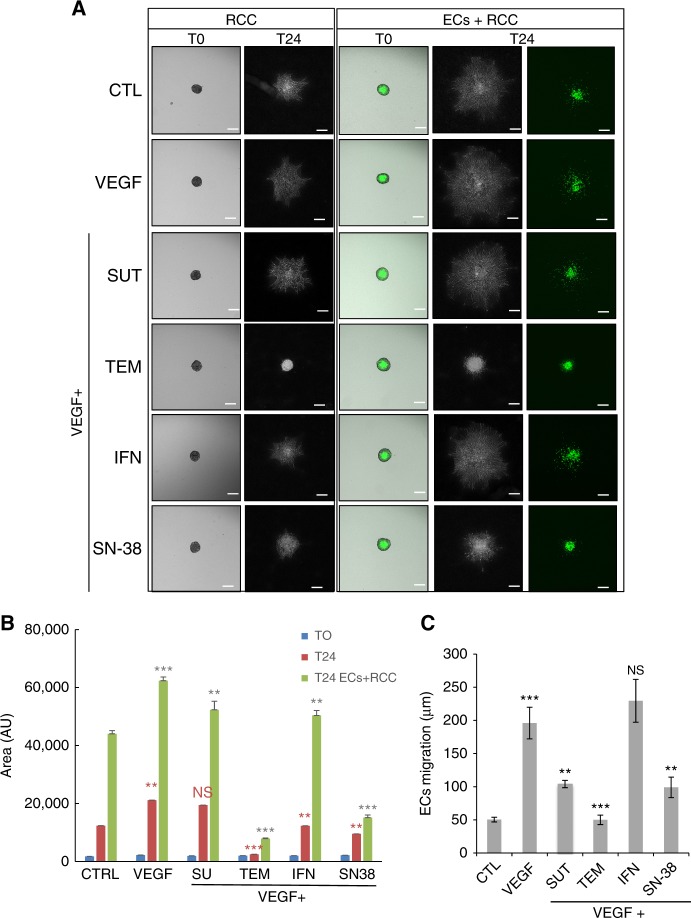
SUT impairs cell invasion and migration in heterotypic spheroids. **a** Representative images of spheroids formed from 786-O (RCC) cells alone at the time of their incorporation into collagen gel (T0) and 24 h later (T24), or RCC + ECs at time 0 and 24 h later. **b** Area of the spheroids was quantified using Image J software (National Institutes of Health). **c** Quantification of ECs migration from spheroids co-cultured with RCC using the measurement of the Euclidean distance of each EC (*n* = 500) from the centre of the spheroid after 24 h of culture. Results are representative of three independent experiments. Values are the mean ± SD of the migration distance in μm. Statistical significance is indicated by the number of asterisks (*)

A confluent ECs monolayer has been used to mimic the vascular endothelium lining all blood vessels. The effect of SUT and TEM on ECs was assessed by analysing tyrosine phosphorylated-proteins as a sensitive readout of the activation of the VEGF/VEGFR signalling pathway. As shown in Fig. 1a, the pattern of VEGF-induced time dependent-tyrosine phosphorylated protein (left panel) was reduced by SUT (middle panel) but not affected by TEM (right panel). The time-dependent-VE-cadherinY^685^-phosphorylation induced by VEGF (Fig. 1b, left panel) was no longer detectable after SUT treatment but remained constant after TEM treatment (Fig. 1b, middle and right panels, respectively). Downregulation of VEGF-induced-VE-cadherin Y^685^ phosphorylation was readily detectable at a SUT concentration as low as 2.5 μM (Fig. 1c, left panel); whereas, the increasing concentrations of TEM (from 0 to 100 ng/mL) had no effect (Fig. 2c, right panel). Because SUT is a TKI, and TEM is a serine-threonine kinase inhibitor, these results are in agreement with the specific effects of these molecules. The destabilisation of ECs after drug treatment was analysed by the release of sVE in the ECs conditioned media. As shown in Fig. 1d, VEGF-induced sVE release was no longer detected with SUT treatment, while it was still detectable with TEM treatment. Other molecules commonly used in cancer treatment, such as interferon α2Α (IFN), irinotecan (IRN), and its active metabolite SN-38, did not affect VEGF-induced sVE release (Fig. 1e). Taken together, these results suggest that the release of sVE from ECs might represent a specific readout of the effects of TKI on ECs, independently of their effects on cancer cells.

### Effect of kidney cancer biotherapies on three dimensional (3D) spheroids of tumour cells and ECs

Because in vitro 3D cell cultures reflect the complex in vivo microenvironment, we next aimed at studying the effects of kidney cancer biotherapies on the sprouting of either one-cell type spheroid containing tumour RCC or a two-cell type co-culture spheroids containing tumour RCC and ECs (labeled with CellTracker). Figure 2a shows representative images of spheroids at the time of their incorporation into collagen gel (T0) and 24 h later (T24). The spheroid area was then quantified at both time of culture upon basal conditions (CTL) or VEGF challenge either alone or in combination with the other indicated drugs. Figure 2b shows that within 24 h the size of the RCC-spheroids expanded sevenfold in CTL conditions (*P* < 0.001) and 12-fold upon VEGF challenge (*P* < 0.001), which is consistent with the presence of VEGF receptors on tumour cells.^24^ TEM, IFN, and SN-38 significantly decreased tumour spheroid expansion, which is in agreement with the anti-tumour effect of these molecules.^25–27^ In contrast, no significant effect of SUT on RCC spheroids was observed, which is in agreement with previous data showing that SUT did not affect RCC proliferation at concentrations that inhibit RTK signalling.^8^

In RCC-ECs 3D structures, spheroids expansion was higher than RCC spheroids in control conditions (areas: 44,038.33 ± 1108.01 vs. 21,156 ± 1106, respectively, *P* < 0.001) suggesting a cooperative effect of both cell types to the spheroid expansion. The expansion of the spheroid was stimulated by VEGF but weakly affected by SU and INF (−20%) and strongly impaired by TEM and SN-38 (−80%). The migration of ECs from the centre of the co-culture spheroids was then determined (Fig. 2c). After 24 h of culture at baseline, the migration of ECs was 50.33 ± 3.51 μm. VEGF alone stimulated the ECs migration by four times (*P* < 0.001) with an average of migration of 196 ± 23.8 μm, while this VEGF-induced ECs migration was partly blocked by SUT (104 ± 5.50 μm, or 53% of VEGF response), and SN-38 (99 ± 15.39 μm, or 50% of VEGF response), and was completely blocked by TEM (50 ± 7.09 μm or control value), but unchanged by IFN 2α (229.66 ± 32.33 μm, or 117% of VEGF response). These results demonstrate that IFN did not impair ECs migration in this 3D co-culture model, but SUT, SN-38 and TEM had a strong inhibitory effect. Taken together, these results indicate that the 3D model is a useful tool to detect the effect of anti-tumour drugs on several cell types including ECs.

### RCC tumour microenvironment induces the release of sVE

**Fig. 3:**
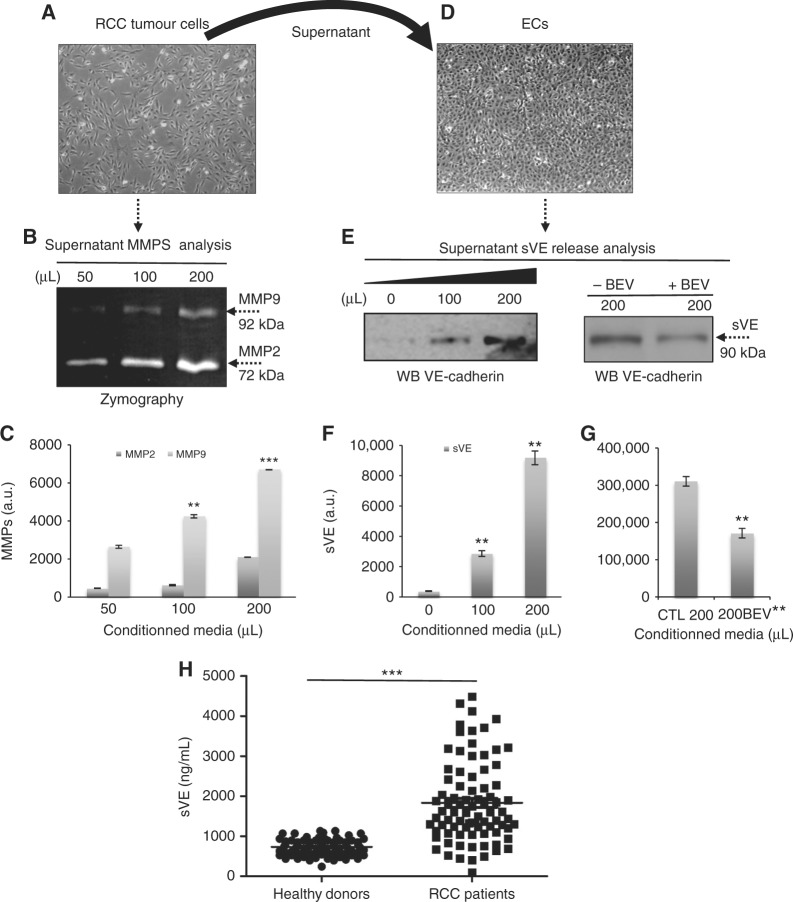
Effect of RCC tumour microenvironment on VE-cadherin cleavage: elevated levels of sVE in mRCC patients:** a** Phase contrast microscopy of RCC cells in culture. **b** Analysis of RCC culture media by zymography showed the presence of MMP2 and MMP9 activities. **c** The relative amounts of MMPs were measured by densitometry of autoradiographs using ImageJ software (National Institutes of Health). **d** Phase contrast microscopy of ECs in culture. **e** Release of sVE by ECs upon RCC conditioned media treatment (20 min) (left panel) alone or RCC conditioned media pre-incubated or not with bevacizumab (BEV) (5 μg/mL) (right panel). **f**, **g** The relative amounts of sVE were measured by densitometry of autoradiographs using ImageJ software (National Institutes of Health). Results are representative of three independent experiments. Values are the mean ± SD. **h** sVE levels in healthy donors (*n* = 96) and mRCC patients from the clinical trial TORAVA (*n* = 115) at diagnosis (Day 0, D0) was performed by ELISA. Statistical significance is indicated by the number of asterisks (*)

The differences in invasion process observed in RCC-ECs co-culture spheroids vs. RCC spheroids, led us to determine whether RCC cells (Fig. 3a) could activate ECs and destabilise adherens junctions by affecting VE-cadherin function. We thus analysed potential RCC (Fig. 3a) secreted inducers of sVE release such as matrix metalloproteinases (MMPs),^28^ using the zymography technique (Fig. 3b). Two MMPs were identified based on their respective molecular weight corresponding to MMP9 (92 kDa) and MMP2 (72 kDa) Analysis of the relative amount of enzyme in the tumour cell conditioned medium showed a dose-dependent expression of MMPs activities with a predominant activity for MMP2 (Fig. 3c). When applied to ECs (Fig. 3d), the tumour cell conditioned media-induced the release of sVE in a dose-dependent manner within 20 min (Fig. 3e, f)). This result is consistent with our previous results showing that TNF-induced VE-cadherin cleavage was mediated by several proteinases.^19^ Because RCC are known to secrete VEGF,^29^ we wondered whether VEGF was involved in this process. Thus the RCC conditioned medium was pre-treated or not with bevacizumab (BEV) for 30 min prior to its addition onto ECs. As shown in Fig. 3g, the release of sVE from ECs was significantly decreased when RCC conditioned medium that was pre-treated with BEV. Taken together, these results are consistent with a role of the tumour microenvironment on the modulation of VE-cadherin functions and ECs behavior in the tumour.

### sVE level is higher in mRCC patients at diagnosis (*n* = 131) than in healthy donors group (*n* = 96)

Because the blood level of sVE has never been reported in mRCC patients, we analysed sVE level by ELISA in a cohort of mRCC patients from the TORAVA and SUVEGIL clinical trials (*n* = 131) that was compared to a group of healthy donors (HD; *n* = 96). The description of mRCC patients is illustrated in Supplementary Table 1. The median level of sVE was significantly higher in mRCC patients (1831 ng/mL [min–max: 90–4484]) than in the HD group (731.0 ng/mL [min–max: 240–1133], *P* < 0.001) (Fig. 3h). As mRCC is a highly vascularised cancer, we hypothesised that sVE might represent a potential new biomarker of the angiogenic processes involved in this type of cancer.

### SUT treatment modulates sVE level in mRCC patients

**Fig. 4:**
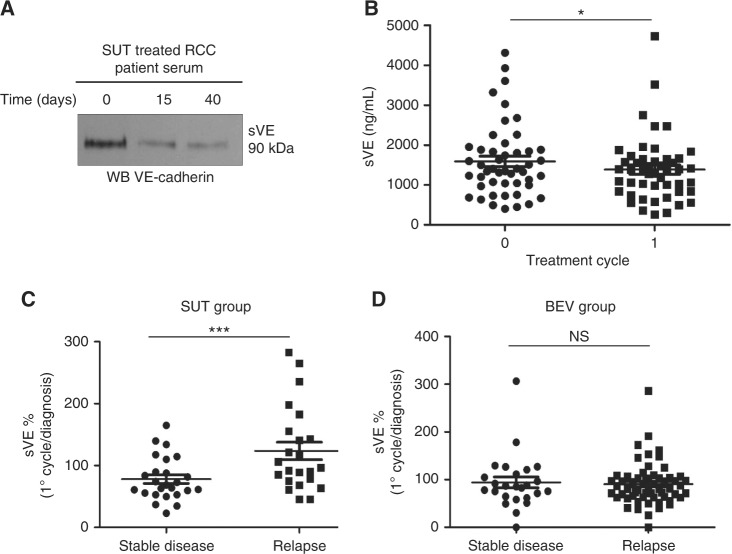
Analysis of sVE- levels in mRCC patients after the first cycle of treatment. **a** Serum from mRCC patient treated with SUT was analysed for sVE-cadherin level using the monoclonal anti-VE-cadherin antibody. **b** sVE levels in mRCC patients after the first cycle of treatment**. c** Variations of sVE expressed as % of the initial concentration at diagnosis (level of sVE after the first cycle/level of sVE at diagnosis × 100) in SUT group with stable disease and relapse. **d** Variations of sVE expressed as % of the initial concentration at diagnosis (level of sVE after the first cycle/level of sVE at diagnosis × 100) in BEV group with stable disease and relapse. Statistical significance is indicated by the number of asterisks (*)

We next determined the effect of SUT treatment on the blood level of sVE in mRCC patients. Figure 4a shows a western blot analysis of serum samples from one patient before treatment (Day 0), after 2 weeks (D15) and after 6 weeks (D40) of SUT treatment. Interestingly, the 90 kDa extracellular domain of VE-cadherin detected in the blood sample is decreased by the treatment for this patient. (Fig. 4a). To further confirm this observation, sVE ELISA was then performed for all mRCC patients from the two clinical trials (TORAVA and SUVEGIL, *n* = 48) at diagnosis and after one cycle of treatment. As a result, we found that the sVE level was significantly decreased after the first cycle of treatment in patients with stable disease (Fig. 4b and Supplementary Figure S2 A) but not in patients who relapsed (Supplementary Fig S1 B). In the BEV group, sVE level after the first cycle was not predictive of the response to the treatment (Figure S2 C, D). We next calculated for each patient the variation of sVE after one cycle of SUT treatment as a % of the initial level at diagnosis (level of sVE after the first cycle/level of sVE at diagnosis × 100). Patients with a stable disease had globally a decreased level of sVE after one cycle of SUT treatment (median ratio 78% corresponding to 22% of decrease) whereas sVE level continued to increase in patients who relapsed after treatment (median ratio 122% corresponding to 22% of increase) (Fig. 4c, *P* = 0.005). sVE variation level was not significantly different in the two groups of patients who received BEV treatment (Fig. 4d) *P* = 0.7469).

### Analysis of sVE variations and clinical outcome

**Fig. 5:**
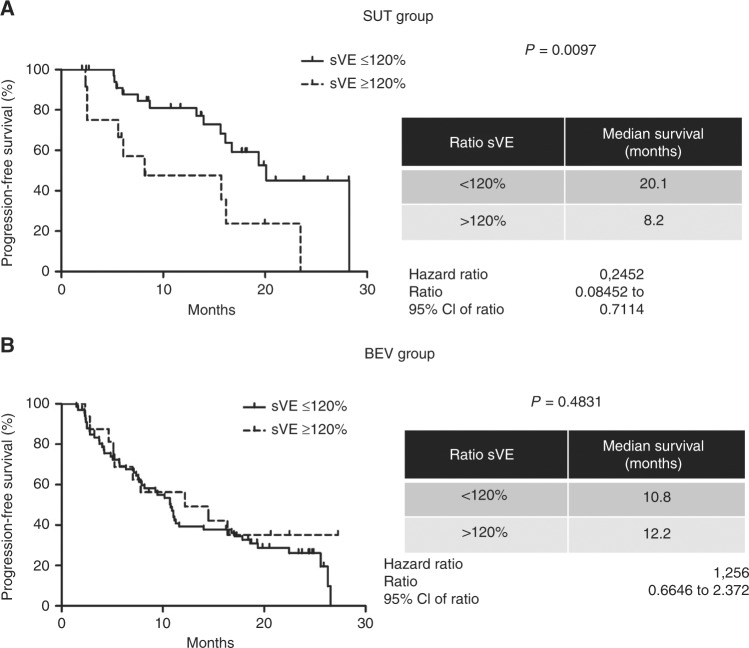
Relationship between sVE-cadherin levels and PFS of mRCC patients: **a**–**c** Kaplan–Meier analysis of PFS of patients with RCC. PFS was calculated from patient subgroups with variations of sVE expressed as % of the initial concentration at diagnosis (level of sVE after the first cycle/level of sVE at diagnosis × 100) that were less or greater than a cutoff ratio at 120%, for SUT group trial (**a**), (**b**) BEV group. Statistical significance (*P-*value) and the time of the median disease free are indicated

sVE variations and progression-free-survival were assessed by the Kaplan–Meier method, using a cutoff of 120% in mRCC patients treated with SUT alone, and in the BEV group. As illustrated in Fig. 5a, patients with an increased level of sVE after SUT treatment (sVE variation >120 %) had a poorer outcome (median PFS 8.2 months) than patients with decreased sVE level (sVE variation <120%—median PFS: = 20.1 months, *P* = 0.0097). We next analysed sVE levels in patients from TORAVA clinical trial treated either with BEV plus IFN or BEV plus TEM. As a result, we found that the variation of sVE levels after the first cycle of treatment was not predictive of the response to treatment (Fig. 5b). The median PFS was 16.7 months and 10.9 months for patients treated with SUT or BEV, respectively (Supplementary Figure S3). Taken together, these results suggest that sVE is a biomarker of response to SUT therapy in mRCC.

## Discussion

Several inhibitors of VEGF-signalling pathways are in clinical use for mRCC management. Because VEGF modulates VE-cadherin structural modifications, in this study we determined the effect of kidney cancer therapies on VE-cadherin functions and its potential clinical interest.

Using a 2D ECs monolayer model to mimic the human endothelium, we studied the early effects of inhibitors of VEGF signalling on VE-cadherin. Among the compounds tested, we demonstrate for the first time that the tyrosine-kinase inhibitor SUT is the only molecule able to block VEGF-induced-sVE release. These observations are in agreement with our previous results obtained using PP2, a Src kinase inhibitor, or Genistein, a large spectrum TKI.^19^ These results are consistent with the specific effects of the inhibitors of VEGF signalling such as TEM, a serine-threonine kinase inhibitor, and IFN, IRN, and SN-38 which are not kinase inhibitors.

By using a 3D homotypic and heterotypic cell culture model to mimic tumour growth we showed that RCC spheroids growth was inhibited by TEM, IFN 2α, and SN-38, which is in agreement with their antitumour activity. Interestingly RCC and ECs co-culture spheroids showed an increased tumour cell invasion, especially upon VEGF stimulation. ECs migration were strongly impaired when VEGF-stimulated spheroids were incubated in the presence of SUT. This result is consistent with a previous study showing that SUT is active in vitro against activated ECs via downregulation of VEGF-Receptor 2.^30^ Interestingly, we found here that the 2D and the 3D models did not provide the same information. Indeed, on the 2D endothelial monolayer, only SUT impaired sVE release which is a process related to ECs migration. In the 3D co-culture spheroids, both SUT, TEM, and SN-38 inhibited ECs migration. These results suggest that the heterotypic spheroid model is a useful model to detect the impact of new anticancer drugs on ECs and to anticipate the potential side effect of new molecules on the vascular system in addition to their anti-tumour activity. The results obtained with the EC monolayers, which reveals the early events involved in VEGF-signalling pathway (20 min), did not identify the potential side effects of these molecules. Nonetheless, it has been reported that after 24 h of treatment, TKIs can inhibit ECs growth, with IC_50_ around 2 μM. TEM also reduced ECs growth by 50%, at concentrations as low as 0.05 or 0.5 nM after 48 or 72 h, respectively.^31^ SN-38 was reported to have anti-angiogenic properties at concentrations ranging from 0.01 to 1 μM (here used at 10 μM).^32^ We used in our study the same concentrations in 2D and 3D cultures (e.g., 50 ng/mL), which are the average concentration of SUT found in blood patients after cycle 1 which is in a direct contact with the endothelium (data not shown). It can be suggested that the long term effect of these concentrations of TKI found in blood might have other side effects on endothelium from healthy vessels. Further studies will be required to determine whether sVE is a biomarker of vascular integrity after biotherapies treatment and possibly a biomarker of toxicities.

**Fig. 6:**
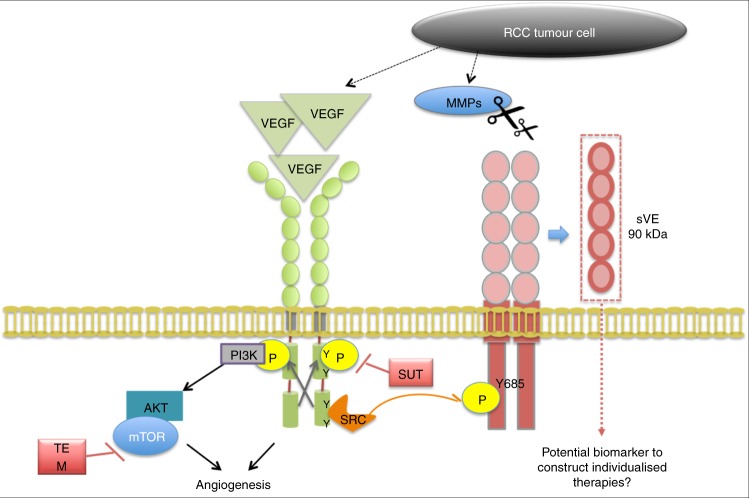
Proposed mechanisms of microenvironment-induced VE-cadherin structural modifications in kidney cancer. This working hypothesis was mainly built from our results obtained using in vitro EC cultures and VEGF as the major actor in angiogenesis.^15, 20^ The involvement of other cytokines in these processes cannot be excluded since we previously showed that TNFα induced sVE release^19^

We have found that tumour cells could activate ECs through VEGF and MMPs secretion, which is in agreement with previous reports (Gialeli et al., 2010) and with our data showing that MMPs are involved in TNFα-induced VE-cadherin cleavage.^19^ It can be assumed that MMPs found in the tumour microenvironment could participate in ECs remodeling by inducing VE-cadherin cleavage, as it was already shown for the cleavage of several cell surface proteins.^33^ Our working hypothesis is illustrated in Fig. 6. Tumour cells secrete cytokines such as VEGF and proteases like MMPs. Upon binding to its receptors, VEGF induces VE-cadherin tyrosine phosphorylation at site Y685 mediated by Src which is a covalent modification that changes VE-cadherin conformation. The protein is thus more sensitive to cleavage by proteases and the soluble form of VE-cadherin is then released. This working model is consistent with the concept of the role of tumour microenvironment in the control of cells contained in a tumour.^34^ The treatment of ECs with SUT inhibits VE-cadherin tyrosine phosphorylation which then inhibits the cleavage and the release of sVE. This is not the case for TEM which is a serine-threonine kinase inhibitor. Thus, the use of sVE as a biomarker might be valuable in constructing individualised therapies for TKI treatments.

Of importance, we found that the level of sVE in mRCC patients at diagnosis is significantly higher than in HD group. This suggests an endothelial junction instability, since sVE is the adhesive part of VE-cadherin that should stay bound to the membrane to ensure vascular integrity.^14^ This result raises the question of the role of the soluble VE-cadherin in cancer. In metastatic breast cancer patients, we previously showed that a decrease of sVE level after 6 weeks of treatment was correlated with good prognosis.^21^ This result suggests that high levels of sVE might be involved in some cell signalling pathways and in tumour activation. This idea is supported by a recent report demonstrating that the extracellular domain of VE-cadherin contains two RGD motifs (specific for human), which are involved in invasion, proliferation and migration of tumour cells through the activation of α2β1 integrin.^35^ Further studies are required to better understand whether sVE in patients possesses tumour promoting functions.

In mRCC, several serum, urinary and plasma biomarkers have recently been described,^36^ including elevated circulating MMPs,^37,38^ as well as neovascularity.^39^ However, our study demonstrates for the first time the presence of sVE in the serum from RCC patients, as well as a SUT-cycle-dependent decrease of sVE level for patients who responded to the therapy. Interestingly, we observed that not all patients exhibited a decrease in sVE level after one cycle of SUT treatment and the reason for this is not known yet. One hypothesis is that the available concentration of the SUT in the bloodstream is different from one patient to another. As shown by therapeutic drug monitoring, the pharmacokinetics of SUT and its active metabolite are subjected to large interindividual variability, especially regarding bioavailability.^40–42^ These variations in the drug concentrations might explain differences in SUT effects on sVE levels in mRCC patients. Then, it would be interesting to study in the future the correlation of sVE levels and the concentrations of drugs in blood to provide information to the clinician in order to potentially adapt the dose of treatment. Furthermore, it would be worth considering a measurement of sVE every 6 weeks instead of one measurement after one cycle of SUT to ensure a better follow-up of mRCC patients.

For the patients treated with a combined therapy, including BEV and TEM or BEV and IFN, we found that the variations of sVE level after one cycle of treatment were not predictive of the response to the treatment. This result does not fit with our in vitro hypothesis (Fig. 6) as we could have expected that any therapy inhibiting VEGF-signalling pathway would have impaired sVE release. However, this result is of major importance as it shows that other potential activated signalling pathways might be involved in tumour progression. Indeed, in a study conducted in a genetic mouse model of neuroendocrine cancer, it was observed that blocking the VEGF pathway by anti-VEGF monoclonal antibody resulted in upregulation of several pro-angiogenicfactors, such as FGF-2, angiopoietin, VEGF, PDGFβ, transforming growth factor α (TGF-α), erythropoietin (EPO), matrix metalloproteinase 1 (MMP-1), epidermal growth factor receptor (EGFR), hepatocyte growth factor receptor (HGFR/cMET), cyclin D1, stromal cell-derived factor 1 (SDF1) and its receptor CXC chemokine receptor 4 (CXCR4).^43^ As BEV is a monospecific drug that blocks only VEGF-A, it does not affect all these actorsactors involved in the remodeling, perfusion, and metabolism of vessels in the tumour, which might participate to VE-cadherin modifications and to tumour escape from anti-VEGF therapy.

SUT and TEM are among the first small molecule inhibitors used in kidney cancer^5^ with some success, but also with some toxicity.^37,44,45^ In the TORAVA study, no benefit was noted in the BEV and TEM combination group^44^ and indeed that study showed that this combination therapy was toxic. Whether or not variations of sVE in patients treated by these combinations are related to toxicities remained to be explored. In contrast to BEV, SUT has the big advantage to inhibit several tyrosine-kinase activities that are overexpressed in cancer, which might explain its better efficacy than other combined therapies.^46^ Further evaluation of sVE-cadherin levels in mRCC patients treated with other TKI is now needed in a prospective study. The quantification of sVE variations for each patient undergoing TKI treatment might provide personalised clinical information about toxicity or responsiveness to therapy, which would fit with the personalised medicine of the future.

## Electronic supplementary material

FigureS1, Figure S2, FigureS3
