# Supplementary material for: The tyrosine-kinase inhibitor sunitinib targets vascular endothelial (VE)-cadherin: a marker of response to antitumoural treatment in metastatic renal cell carcinoma
Source: Br J Cancer. 2018 Mar 22;118(9):1179–88. doi: 10.1038/s41416-018-0054-5 (PMC5943344; doi:10.1038/s41416-018-0054-5)
Supplement: Supplementary file 1 — FigureS1, Figure S2, FigureS3 [file 41416_2018_54_MOESM1_ESM.pptx]

## Slide 1
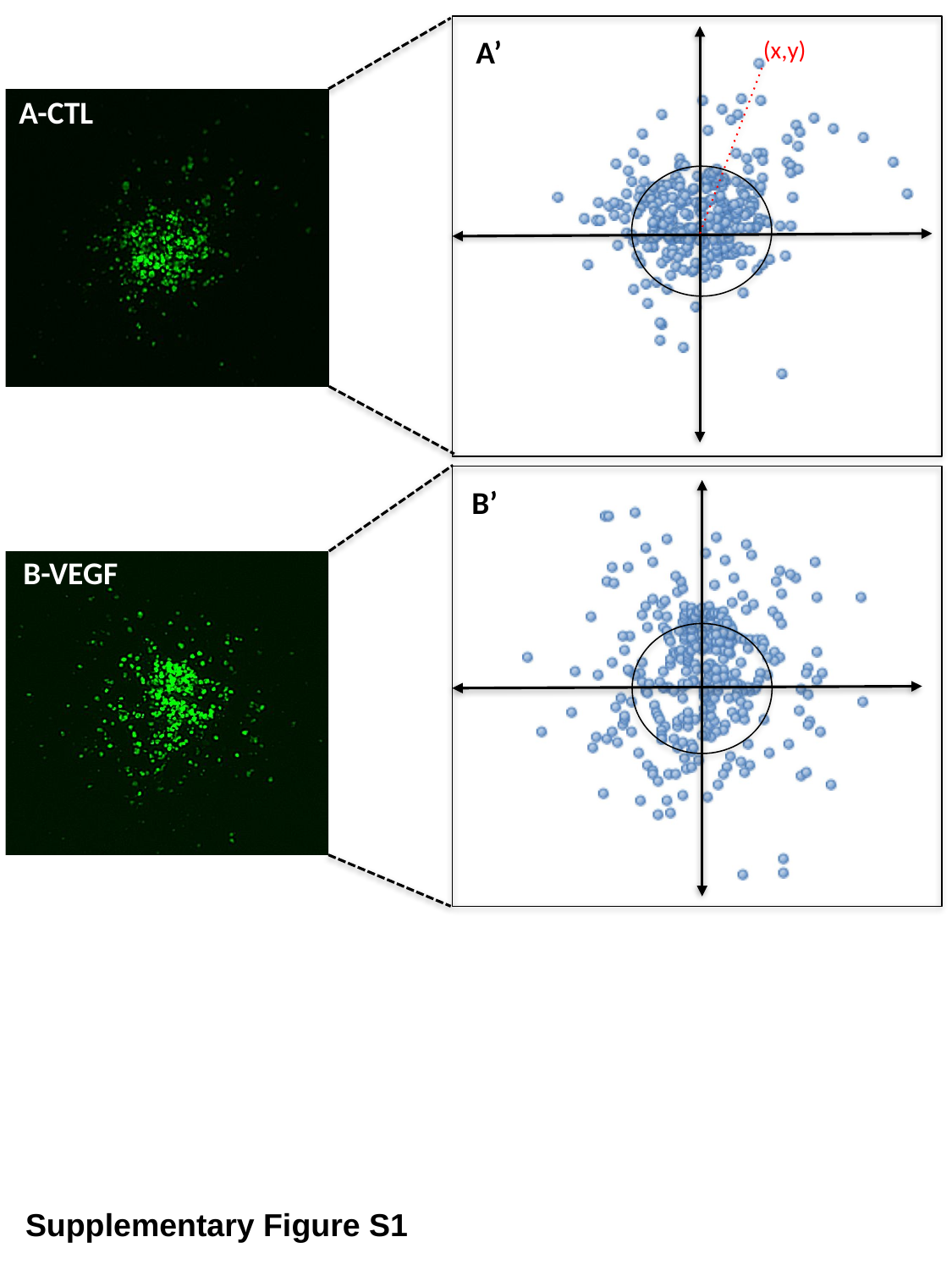

A’
(x,y)
A
A-CTL
B’
B-VEGF
Supplementary Figure S1

## Slide 2
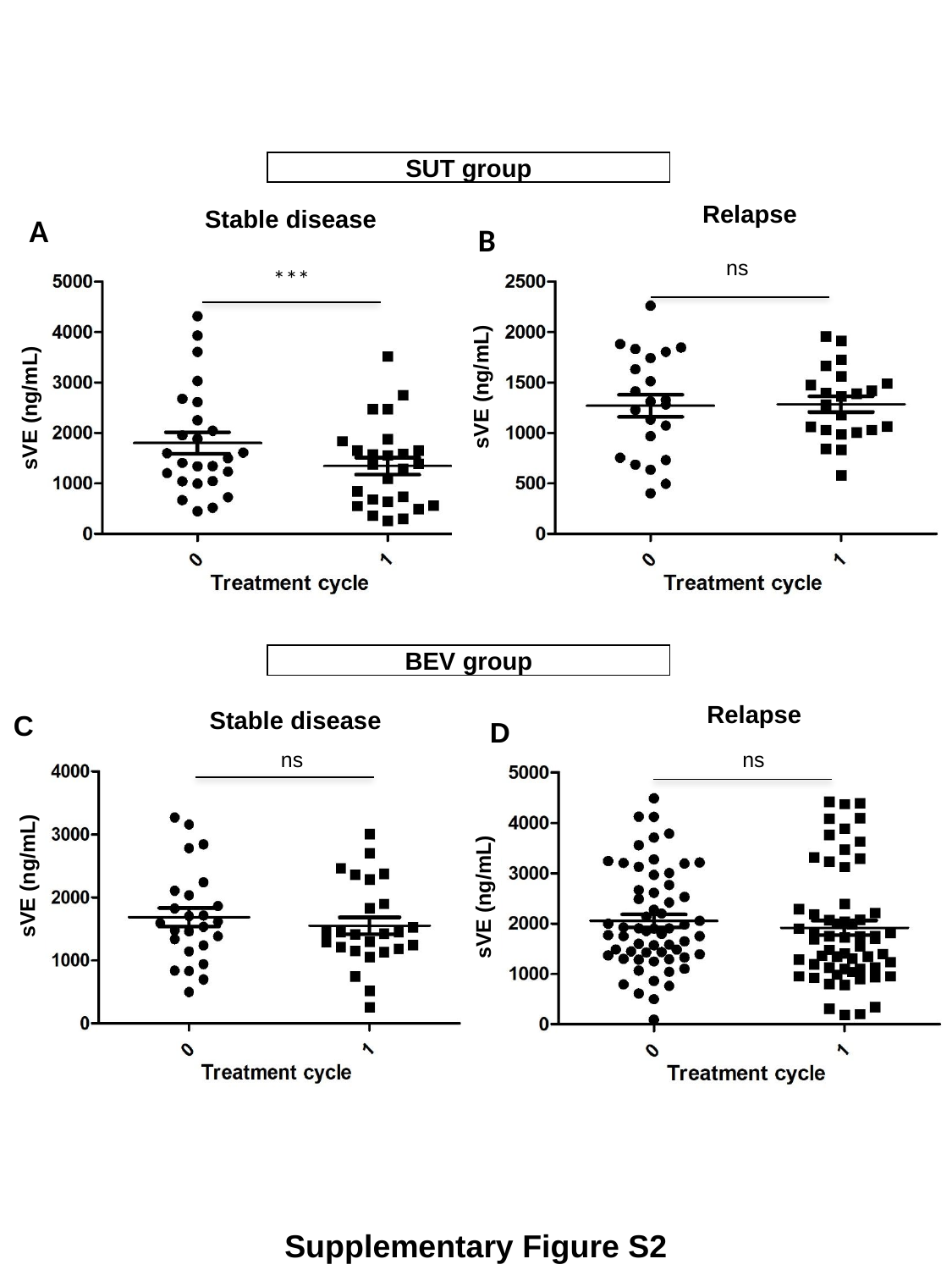

SUT group
Relapse
Stable disease
A
B
ns
***
sVE (ng/mL)
sVE (ng/mL)
BEV group
Relapse
Stable disease
C
D
ns
ns
sVE (ng/mL)
sVE (ng/mL)
Supplementary Figure S2

## Slide 3
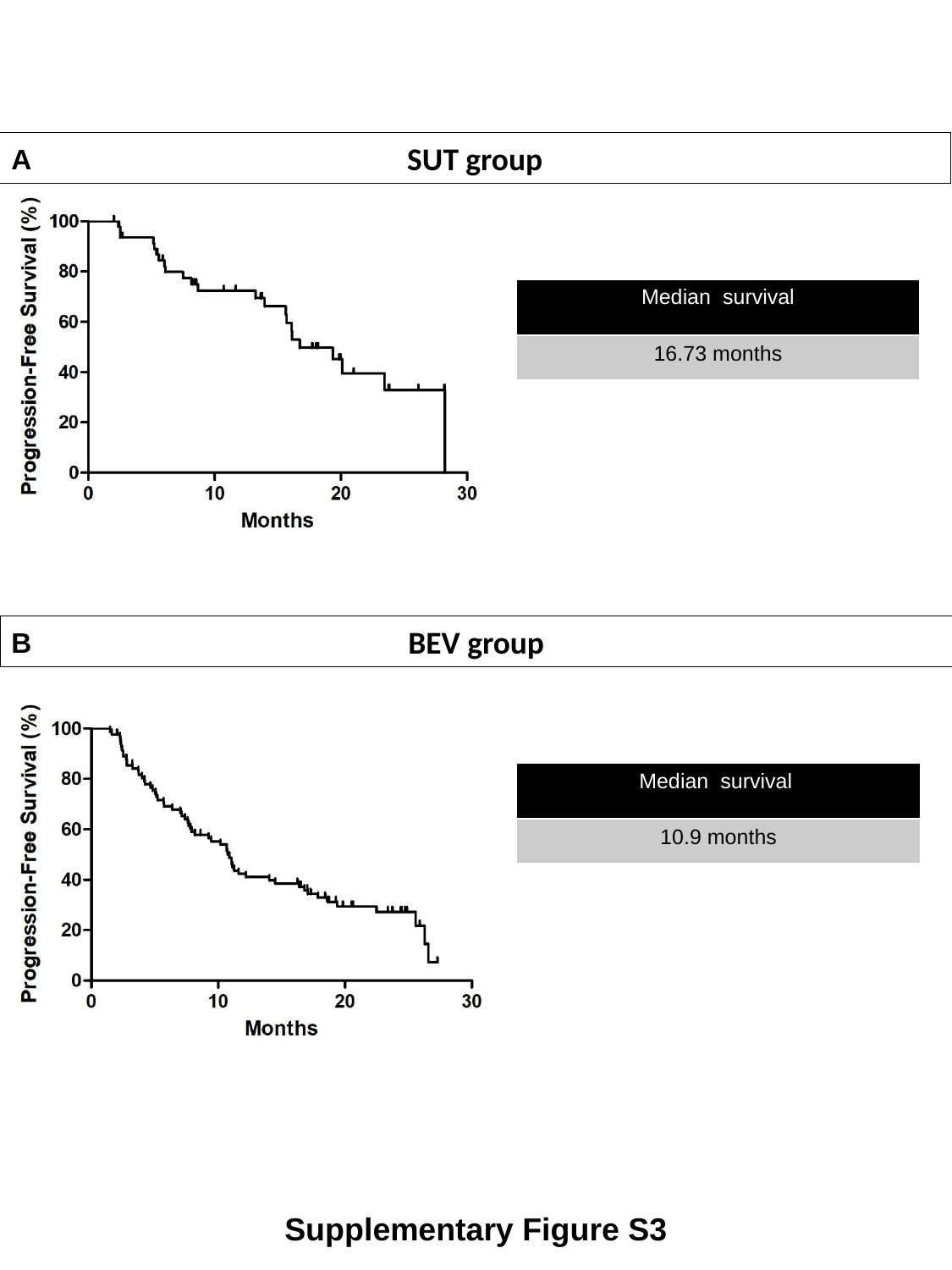

SUT group
A
| Median survival |
| --- |
| 16.73 months |
BEV group
B
| Median survival |
| --- |
| 10.9 months |
Supplementary Figure S3

## Slide 4
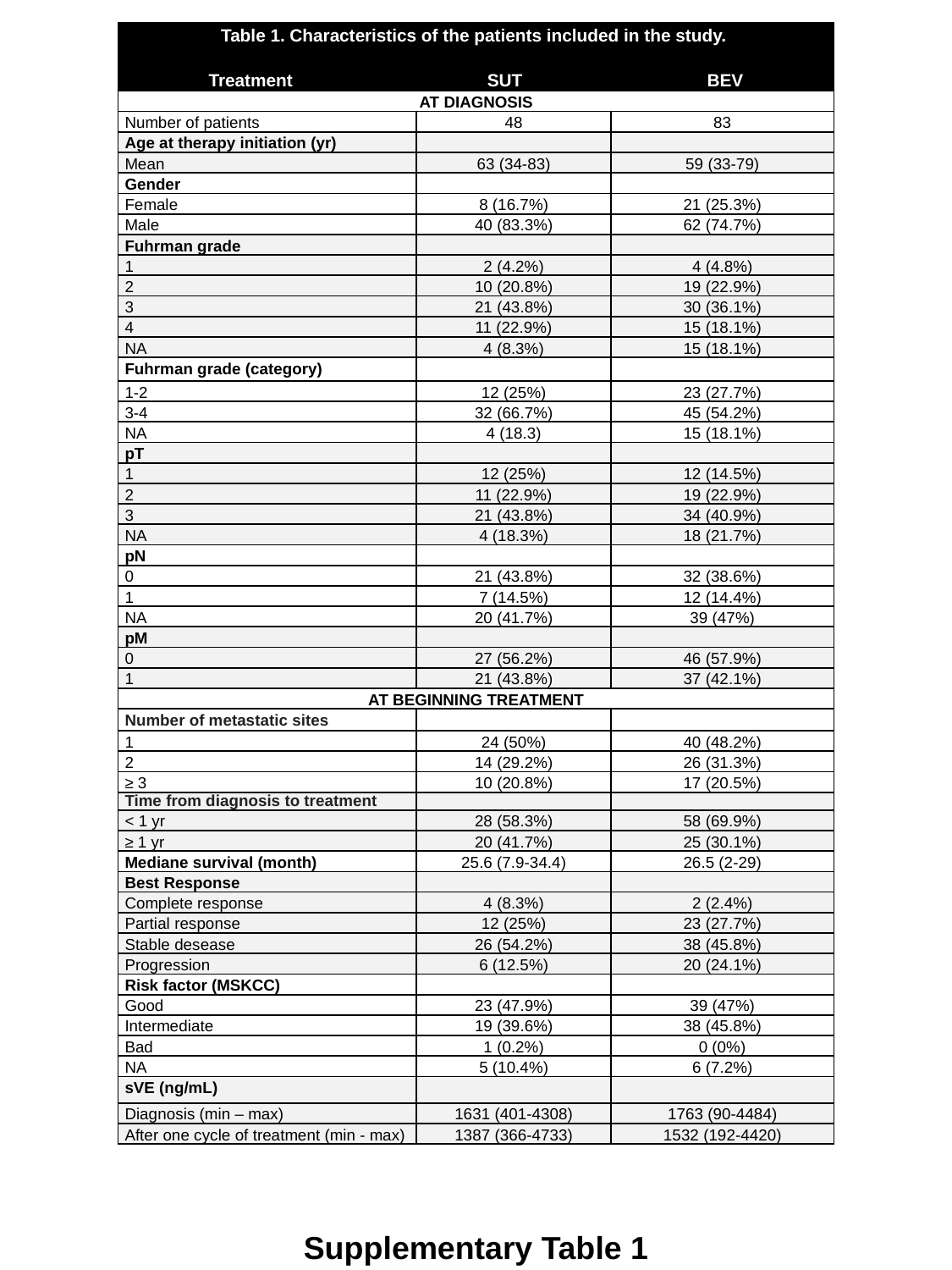

| Table 1. Characteristics of the patients included in the study. | | |
| --- | --- | --- |
| Treatment SUT BEV | | |
| AT DIAGNOSIS | | |
| Number of patients | 48 | 83 |
| Age at therapy initiation (yr) | | |
| Mean | 63 (34-83) | 59 (33-79) |
| Gender | | |
| Female | 8 (16.7%) | 21 (25.3%) |
| Male | 40 (83.3%) | 62 (74.7%) |
| Fuhrman grade | | |
| 1 | 2 (4.2%) | 4 (4.8%) |
| 2 | 10 (20.8%) | 19 (22.9%) |
| 3 | 21 (43.8%) | 30 (36.1%) |
| 4 | 11 (22.9%) | 15 (18.1%) |
| NA | 4 (8.3%) | 15 (18.1%) |
| Fuhrman grade (category) | | |
| 1-2 | 12 (25%) | 23 (27.7%) |
| 3-4 | 32 (66.7%) | 45 (54.2%) |
| NA | 4 (18.3) | 15 (18.1%) |
| pT | | |
| 1 | 12 (25%) | 12 (14.5%) |
| 2 | 11 (22.9%) | 19 (22.9%) |
| 3 | 21 (43.8%) | 34 (40.9%) |
| NA | 4 (18.3%) | 18 (21.7%) |
| pN | | |
| 0 | 21 (43.8%) | 32 (38.6%) |
| 1 | 7 (14.5%) | 12 (14.4%) |
| NA | 20 (41.7%) | 39 (47%) |
| pM | | |
| 0 | 27 (56.2%) | 46 (57.9%) |
| 1 | 21 (43.8%) | 37 (42.1%) |
| AT BEGINNING TREATMENT | | |
| Number of metastatic sites | | |
| 1 | 24 (50%) | 40 (48.2%) |
| 2 | 14 (29.2%) | 26 (31.3%) |
| ≥ 3 | 10 (20.8%) | 17 (20.5%) |
| Time from diagnosis to treatment | | |
| < 1 yr | 28 (58.3%) | 58 (69.9%) |
| ≥ 1 yr | 20 (41.7%) | 25 (30.1%) |
| Mediane survival (month) | 25.6 (7.9-34.4) | 26.5 (2-29) |
| Best Response | | |
| Complete response | 4 (8.3%) | 2 (2.4%) |
| Partial response | 12 (25%) | 23 (27.7%) |
| Stable desease | 26 (54.2%) | 38 (45.8%) |
| Progression | 6 (12.5%) | 20 (24.1%) |
| Risk factor (MSKCC) | | |
| Good | 23 (47.9%) | 39 (47%) |
| Intermediate | 19 (39.6%) | 38 (45.8%) |
| Bad | 1 (0.2%) | 0 (0%) |
| NA | 5 (10.4%) | 6 (7.2%) |
| sVE (ng/mL) | | |
| Diagnosis (min – max) | 1631 (401-4308) | 1763 (90-4484) |
| After one cycle of treatment (min - max) | 1387 (366-4733) | 1532 (192-4420) |
Supplementary Table 1
